# Generation of functional fat organoid from rat superficial fascia

**DOI:** 10.1080/21623945.2022.2072446

**Published:** 2022-05-13

**Authors:** Yanfei Zhang, Yuanyuan Zhang, Yingyue Dong, Tongsheng Chen, Guoheng Xu

**Affiliations:** a Department of Physiology and Pathophysiology, Peking University School of Basic Medical Sciences, and Peking University Center for Obesity and Metabolic Disease Research, Beijing, Peking, China; b Key Laboratory of Functional and Clinical Translational Medicine, Department of Physiology, Xiamen Medical College, Xiamen, Fujian, China

**Keywords:** Superficial fascia, fascial preadipocyte, 3D hydrogel, fat organoid, adipogenesis, adipose tissue

## Abstract

The organoid is a 3D cell architecture formed by self-organized tissues or cells in vitro with similar cell types, histological structures, and biological functions of the native organ. Depending on the unique organ structures and cell types, producing organoids requires individualized design and is still challenging. Organoids of some tissues, including adipose tissue, remain to generate to be more faithful to their original organ in structure and function. We previously established a new model of the origin of adipose cells originating from non-adipose fascia tissue. Here, we investigated superficial fascia fragments in 3D hydrogel and found they were able to transform into relatively large adipocyte aggregates containing mature unilocular adipocytes, which were virtually “fat organoids”. Such fascia-originated fat organoids had a typical structure of adipose tissues and possessed the principal function of adipose cells in the synthesis, storage, hydrolysis of triglycerides and adipokines secretion. Producing fat organoids from superficial fascia can provide a new approach for adipocyte research and strongly evidences that both adipose tissues and cells originate from fascia. Our findings give insights into metabolic regulation by the crosstalk between different organs and tissues and provide new knowledge for investigating novel treatments for obesity, diabetes and other metabolic diseases.

**Abbreviations**: 3D: three dimensional; ASC: adipose-derived stromal cells; C/EBP: CCAAT-enhancer-binding protein; EdU: 5-ethynyl-2-deoxyuridine; FABP4: fatty acid-binding protein 4; FAS: fatty acid synthase; FSCs: fascia-derived stromal cells; Plin1: perilipin-1; Plin2: perilipin-2; PPARγ: peroxisome proliferator-activated receptor γ; WAT: white adipose tissue

## Introduction

The generation of organs or tissues *in vitro* has been a long-standing dream. Solid organs and tissues in high mammals have a large mass and volume and have vigorous nutritional demand, hence increasing the difficulty of their long-time survival *in vitro*. Although many difficulties remain in the reconstruction of organs *in vitro*, except for tumour tissues with strong proliferation abilities [1], self-organizing 3D organoids of various types of organs and tissues have received considerable attention and achieved great progress over the last 10 years [2,3]. Recently, constructions of organoids include retina, brain, prostate, kidney, lung, liver, pancreas, stomach, and intestine have been reported [2–4]. However, because organs have unique histology of structure, cell type, and growth characteristic, the construction of organoids is still individualized and challenging. Many organoids remain to be generated with more faithful tissue-like structures and functions of their native organs, such as adipose tissue, although several studies have concentrated on the construction of adipose-tissue organoids [5–12].

Fascia is a mesoderm-derived connective tissue that is capable of osteogenic, chondrogenic, and adipogenic differentiation *in vitro* [13–16]. Previously, our laboratory showed that the superficial fascia of rats contains fibroblasts, mast cells, mature adipocytes, and abundant lineage-committed preadipocytes, which are always distributed along the vasculature and present active adipogenesis during the developmental periods. Therefore, we proposed a novel theory of the fascial origin of adipocytes [13,17]. We identified that mast cells and their granule heparin act as endogenous regulatory factors that initiate fascial adipogenesis [18], and also, we revealed that fascial adipocytes showed unique cytological and functional properties that maintained high basal lipolysis but were not sensitive to catecholamines as compared with subcutaneous and visceral adipocytes [19]. These results indicate that adipose cells can originate from the superficial fascia, which represents a novel origin of adipocytes with different cytological, functional and developmental features [13,17–19].

Fascia-derived fat organoids represent an invaluable tool for *in vitro* and *ex vivo* studies simulating *in vivo* environments and have broad application prospects in regenerative medicine, plastic surgery, and personalized treatment. In this study, we investigated the generation of functional 3D fat organoids originating from superficial fascia. Because the fascia in rats, different from that in humans, is easily separated from adjacent tissues and contains fewer mature adipocytes [13], superficial fascia of rat hindlimbs was selected as an initial experimental material. We found that fascia fragments in the 3D hydrogel were able to transform into organized adipocyte aggregates that contain adipogenic progenitors, early-differentiated adipocytes with numerous tiny lipid droplets, and late-differentiated unilocular mature adipocytes with a single, large lipid droplet, which were consistent with the morphology of mature adipose cells and adipose tissue *in vivo*. Such fascia-originated adipocyte aggregates were virtually fat organoids, featured by typical structure of adipose tissue and adipocytes and possessed the principal function of adipose cells in the synthesis, storage, and hydrolysis of triglycerides. Also, these data provide strong evidence for the fascial origin of fat cells and fat organoids, which may strengthen the understanding of metabolic regulation involving crosstalk between multiple different organs and tissues, such as adipose tissue and fascia.

## Results

### Adipocytes and adipogenic progenitors in superficial fascia

**Figure 1.**
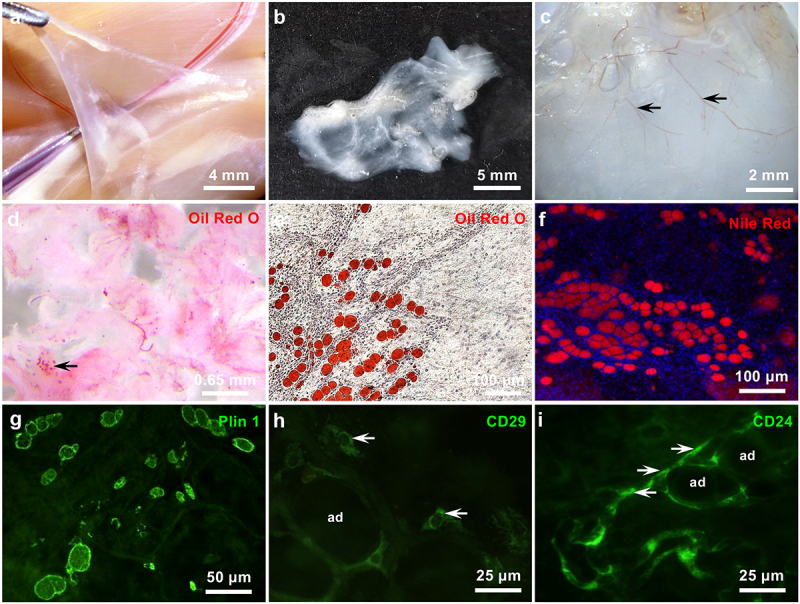
**Adipocytes and adipogenic progenitors in superficial fascia**. Five-week-old male rats were used. (a) The gross anatomy of the hindlimb superficial fascia situated above the skeletal muscles, below which was the vein, saphenous artery and nerve of the muscles deep fascia. (b, c) Stereomicroscopy vision of freshly dissected fascia. Arrows are vasculature. (d) Superficial fascia was mined into 1- to 3- mm^3^ fragments and stained with Oil-red O. Arrows are adipocytes in fascia. (e, f) Whole-mounted superficial fascia was stained with Oil-red O and Nile Red. Nuclei were stained by Hoechst 33258 (blue). (g–i) Whole-mounted superficial fascia was stained by antibodies against Plin1, CD29 and CD24, separately labelling differentiated adipocytes and adipogenic progenitors. Arrows are CD29- and CD24-positive precursors.

The superficial fascia of 5-week-old rats was viscoelastic and semitransparent and was easily isolated from the deep fascia and muscle epimysium *in vivo* (Figure 1(a)). After isolation, fascial sheets were white and jelly-like with abundant vasculature (Figure 1(b,c)) and were whole-mounted on glass slides. When minced and stained with Oil-red O, fascia fragments appeared light red (Figure 1(d)), due to the present of a few number of adipocytes in some regions of fascia. For microscopy observations, the whole-mounted superficial fascia was positively stained with Oil-red O, Nile Red, and perilipin 1 (Plin1) (Figure 1(e–g)), an adipocyte-specific protein coating lipid droplets mainly in adipocytes or steroidogenic cells [20,21], thus showing a typical mature adipocytes phenotype. Superficial fascia was immunostained with adipogenic progenitor markers CD29 or CD24 [22], and CD29-positive cells were typically larger and scattered throughout the whole-mounted fascia (Figure 1(h)). CD24-positive precursor cells appeared smaller and spindly and were located over the inferior blood vessels surrounding mature adipocytes (Figure 1(i)). As expected, fascial cells could also spontaneously and inducibly accumulate a considerable amount of cytoplastic lipid droplets in conventional 2D culture [13].

### Adipogenesis of the superficial fascia in the hydrogel

**Figure 2.**
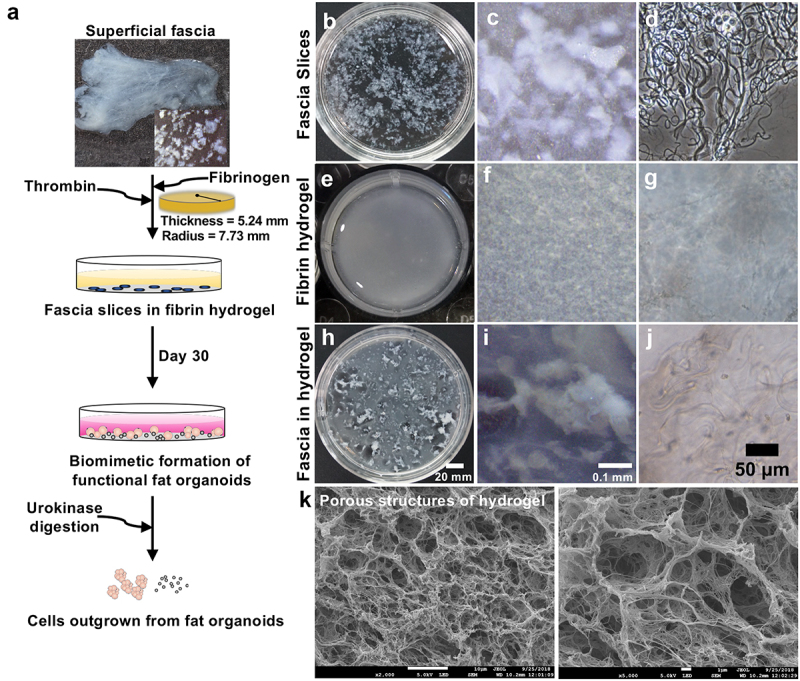
**Fibrin hydrogel-supported 3D tissue culture of superficial fascia**. (a) Summary figure representing an overall process for developing fat organoids of superficial fascia in the 3D fibrin hydrogel. (b–j) The superficial fascia was minced into 1- to 3- mm^3^ sized fragments, evenly suspended in a fibrinogen and thrombin mixture solution to obtain 3D hydrogel-encapsulated fascia fragments. Sliced superficial fascia fragments (b, c); hemp-like elastic fibres in the superficial fascia, attached to fascial cells and fascial stem cells (d); completely polymerized 3D fibrin hydrogel (e–g); hydrogel-encapsulated fascia fragments (h, i); and fascial cells beginning to emerge from the fascial fibrous networks and proliferate (j). SEM image of porous microstructures of the hydrogel (k).

Hydrogel-based 3D tissue culture of the superficial fascia supported direct cell outgrowth and proliferation in the fibrin matrix (Figure 2(a)). When dissected, superficial fascia appeared as a pale jelly-like structure, with elastic and collagen fibres clearly discernible (Figure 2(b–d)). Then, the superficial fascia was minced into small fragments, and suspended in a mixture of fibrinogen and thrombin solution to obtain 3D hydrogel-encapsulated fascia fragments (Figure 2(e–j)). Scanning electron microscopy (SEM) showed a porous network structure of the hydrogel scaffolds. The mechanical properties of the hydrogel enabled its manipulation with surgical tweezers for placement on glass slides for further research (Figure 2(k)).

**Figure 3.**
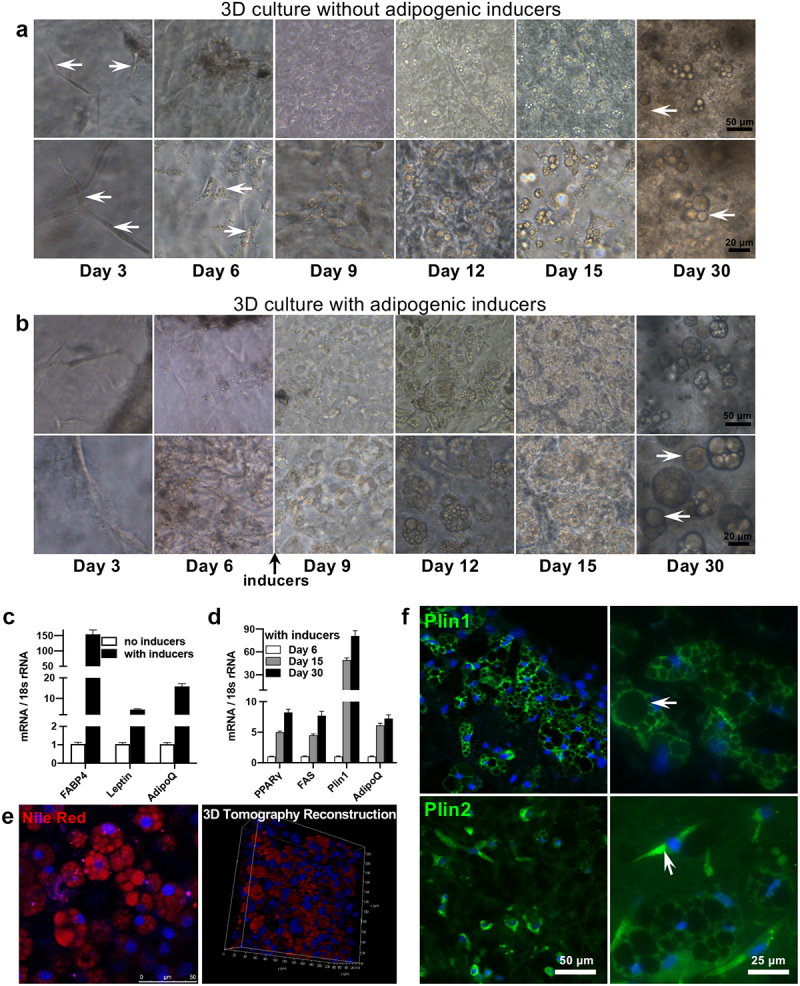
**Adipogenic differentiation of the superficial fascia in the 3D fibrin hydrogel**. (a) Phase-contrast photographs of the cells outgrown from superficial fascia fragments on day 3 to 30 but without adipogenic inducers. The outgrown cells exhibited spindle-like morphology (arrow) on day 3, small lipid droplets (arrow) appeared on day 6, and then the size and number of lipid droplets gradually increased. Day 9 to 30, the outgrown cells spontaneously accumulated a number of large lipid droplets. On day 9, the lipid droplets were clearer and more recognizable. On day 30, mature unilocular adipocytes containing a single, large lipid droplet appeared and crowded into the organized aggregates of adipocytes (arrow), which were ‘fat organoids’. (b) Photographs of outgrown cells from the superficial fascia with adipogenic inducers. Induced cells showed significantly more and larger cytoplasmic lipid droplets, including unilocular lipid droplets (arrow) with diameters from 10 to 20 μm. (c) The mRNA levels of FABP4, leptin, and adiponectin (AdipoQ) upon adipogenic induction. (d) Gene expression of fascial adipocytes in the hydrogel at different differentiated time points with adipogenic inducers. Data are mean ± SEM. (e) Nile Red staining of the 3D fascial architectures in the hydrogel. Microscopy view and 3D reconstructed imaging (width 240.05 μm, height 240.05 μm, depth 80.50 μm) of fascia in the fibrin showed numerous differentiated adipocytes containing lipid droplets. (f) Immunofluorescence assay of Plin1 and Plin2 labelling late- and early-differentiating adipocytes (arrow), respectively. Nuclei were stained by Hoechst 33258 (blue). AdipoQ, adiponectin; FABP4, fatty acid binding protein 4; PPARγ, peroxisome proliferator-activated receptor γ; FAS, fatty acid synthase.

On day 3, outgrown cells showed elongated and branched cytoplasm in multiple directions, such as a spindle-like appearance (Figure 3(a)). On day 6, the proliferated cells reached confluency, and small lipid droplets appeared (Figure 3(a)). Thereafter, the size and number of lipid droplets gradually increased. On day 9, the lipid droplets were clearer and more recognizable (Figure 3(a)). On day 30, mature adipocytes containing large lipid droplets or even unilocular lipid droplets appeared and crowded into the organized aggregates of adipocytes (Figure 3(a)), which were actually the ‘fat organoids’. With adipogenic inducers, induced cells showed significantly more and larger cytoplasmic lipid droplets (Figure 3(b)).

As expected, differentiated fascial adipocytes expressed significantly higher mRNA levels of fatty acid-binding protein 4 (FABP4), leptin and adiponectin, which are two major adipocyte-specific adipokines (Figure 3(c)). Peroxisome proliferator-activated receptor γ (PPARγ), fatty acid synthase (FAS), Plin1, adiponectin genes were all upregulated during adipogenic differentiation in a time-dependent manner, in parallel with an increased lipid accumulation and adipocyte maturity (Figure 3(d)). Nile Red staining showed multiple adipocyte-spheroid architectures densely distributed over the whole hydrogel (Figure 3(e)). Plin1 exclusively coats lipid droplets and participates in regulating adipocyte differentiation [20]. Plin2 is suggested to regulate fatty acid mobilization and lipid droplet formation [23]. Immunohistochemical staining showed that adipocyte spheroids were positively labelled with Plin1, showing the typical characteristic of late-differentiated adipocytes, and with Plin2, the early-differentiated protein, thus suggesting an early differentiated adipocyte phenotype (Figure 3(f)). These results indicate that superficial fascia fragments were highly capable of adipogenesis and could form organized adipocyte aggregates in the hydrogel.

### Formation of fascial fat organoids

**Figure 4.**
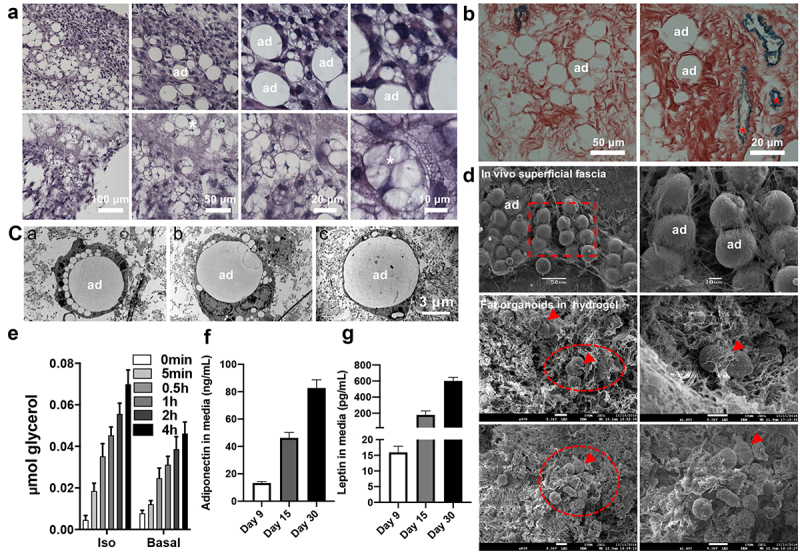
**Formation of fascial fat organoids**. (a) H&E staining showed numerous differentiation stages of adipocytes, including fully mature unilocular adipocytes (ad) and partially differentiated multilocular adipocytes (white asterisk), gathered together into fat organoids. (b) Victoria Blue-Ponceau staining showed mature adipocytes (ad) containing large lipid droplets, microvessels (blue, red asterisk), and collagenous material (red). (c) TEM micrographs showed emerging mature adipocytes (ad) with a large white vacuole and few tiny lipid droplets with diameter approximately 5 μm and fully mature unilocular adipocytes containing a single, large vacuole, with mean diameter from 10 to 20 μm. (d) SEM images show the superficial fascia *in vivo* and fascial fat organoids in the hydrogel. The amplified square box areas represent adipocytes of superficial fascia *in vivo*. Arrowheads refer to adipocytes. Oval boxes show fat organoids, with average volume 100 to 300 μm^3^. (e) Fat-organoids lipolysis function. Glycerol release from fat organoids stimulated with or without 1 μM isoproterenol (Iso, F, G) Adipokine secretions of fat organoids were assayed by enzyme-linked immunosorbent assay. Data are mean ± SEM.

As shown in Figure 3, successful fat organoids were formed by 3D culture of the superficial fascia for 30 days with or without adipogenic inducers. H&E (Figure 4(a)) and Victoria blue-Ponceau staining (Figure 4(b)) revealed that fat organoids contained dense mature adipocytes, abundant collagens, a few of elastic fibres, and small blood vessels (asterisk), resembling adipose tissue *in vivo*. TEM showed emerging mature adipocytes with a large white vacuole and few tiny lipid droplets with diameter

approximately 5 μm and fully mature unilocular adipocytes with a single, large vacuole (Figure 4(c)), with average diameter 10 to 20 μm. The size was less than that of the superficial fascia adipocytes *in vivo*, which were approximately 35 μm at 5 to 6 weeks [19] and shuttled through fibrous networks (Figure 4(d)). SEM observations of the organized mature adipocyte aggregates revealed ‘fat organoids’ (dotted boxes), with average volumes from 100 to 300 μm^3^ (Figure 4(d)).

To evaluate the functional features of fat organoids, we assayed lipolysis reaction and adipokines secretion. Upon stimulation of catecholamine isoproterenol, glycerol release from fat organoids was increased time-dependently (Figure 4(e)). After the removal of isoproterenol for 10 min, glycerol re-release remained increased time-dependently, exhibiting a typical adipocyte lipolysis in response to hormonal stimulation (Figure 4(e)). Adiponectin and leptin are two major adipokines secreted mainly by adipose tissue [24]. As shown in Figure 4(f,g), fascial fat organoids showed increased adiponectin and leptin in conditioned medium with increasing culture time.

### Cytological phenotypes of the cells outgrown from fat organoids

**Figure 5.**
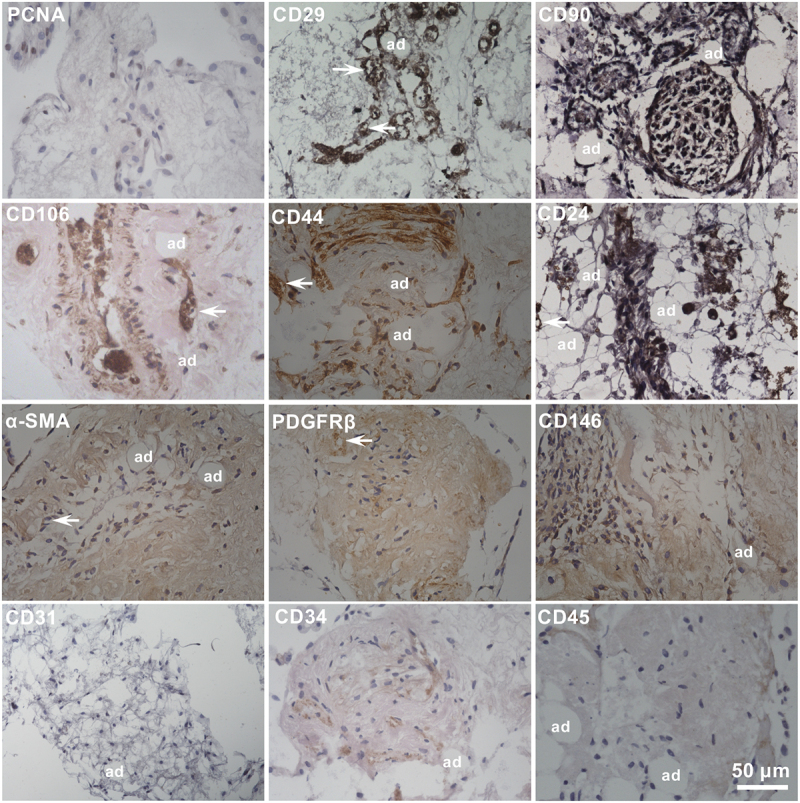
**Immunophenotype of fascial fat organoids**. The fascia fragments were embedded in the hydrogel and cultured for 30 days without adipogenic inducers. Outgrown cells always expressed stromal cells markers (CD29, CD90, CD106, CD44, CD24) and pericyte markers (α-SMA, PDGFRβ, CD146) residing along microvessels (arrow) and mature adipocytes (ad). However, markers of endothelial cells CD31and CD34, and haematopoietic cells CD45, were almost not detected.

To determine the *in situ* immunohistochemical profiles of outgrown cells, we stained sections that contained outgrown cells, cultured fascial tissue fragments and hydrogel (Figure 5). Given that adipogenic progenitors always maintained an active proliferative state, we first measured the proliferation of outgrown cells. Approximately 50% of the outgrown cells expressed PCNA (Figure 5), and 50% to 80% of the outgrown cells incorporated EdU, whose positive expression was higher near the upper layer of the fibrin matrix possibly because of the more abundant nutrient and gas exchange (Figure S1). These results suggest that the most of the outgrown cells were from cell proliferation and division *in vitro* and not simple *in situ* migration of resident cells in fascia fragments. For immunostaining, the outgrown cells displayed the expression profiles of stromal cell markers, namely CD29, CD90, CD106, CD44, CD24, α-SMA, PDGFRβ and CD146, with the first five labelling adipogenic progenitors [25] and the last three labelling pericytes [26]. However, outgrown cells rarely expressed endothelial markers, namely CD31, and CD34, and the haematopoietic marker CD45 (Figure 5).

Consistent with the histochemical results, flow cytometry revealed that the outgrown cells presented immunophenotypic profiles similar to those of stromal adipogenic progenitors and vascular pericytes (Table S3). The outgrown cells contained a substantial cell subset expressing CD29 (99.21%), CD44 (63.06%), CD90 (90.96%), or CD106 (53.74%) but a relatively small subset expressing CD24. Similarly, outgrown cells preserved the pericyte markers α-SMA (30.26%) and PDGFRβ (43.64%). In contrast, the cells were negative for the endothelial marker CD31 (0.13%) or haematopoietic marker CD45 (0.24%).

### Adipogenic differentiation of cells outgrown from fat organoids

**Figure 6.**
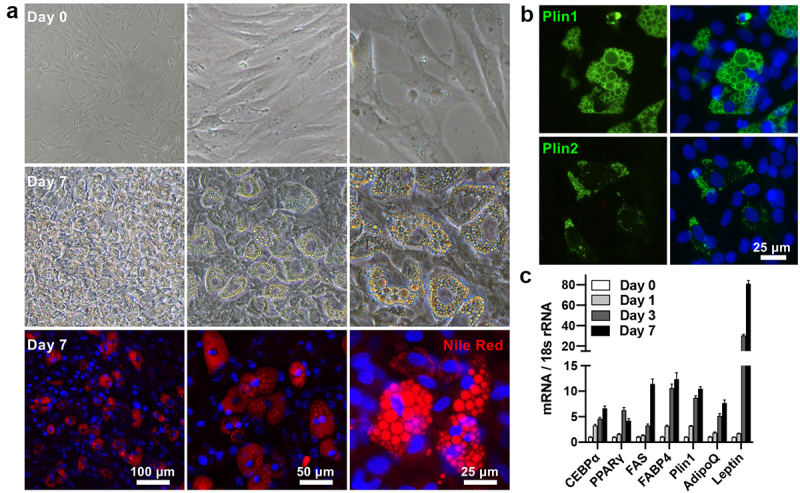
**Adipogenic differentiation of cells outgrown from fat organoids**. (a) *In vitro* differentiation potential of the outgrown cells with adipogenic inductions. Outgrown cells isolated from fat organoids were treated with adipogenic inductions for 2 days and cultured for another 5 days with maintenance medium. On day 7, differentiated adipocytes containing lipid droplets were stained with Nile Red. Induced cells showed an accumulation of cytoplasmic triglycerides. (b) Immunofluorescence of Plin1 and Plin2. Nuclei were stained by Hoechst 33,258 (blue). (c) qRT-PCR analysis of the mRNA expression of adipogenic-related factors in differentiated outgrown cells from day 0 to 7. Data are mean ± SEM. C/EBP, CCAAT-enhancer-binding protein.

After 3D tissue culture of the superficial fascia for 30 days, the fascial outgrown cells were successfully recovered by using the serine protease urokinase, which can transmute plasminogen to plasmin, selectively degrading fibrin [27]. Harvested cells were plated in conventional 2D culture. The day that the outgrown cells appeared confluent was considered day 0. After 1-week induction, approximately 50% of outgrown cells accumulated numerous cytoplasmic lipid droplets, positively stained by Nile Red and Plin1 but scarcely stained by Plin2 (Figure 6(a,b)). The mRNA levels of the adipogenic-related factors CCAAT-enhancer-binding protein α (CEBPα), PPARγ, FAS were significantly increased in differentiating outgrown cells from day 0 to 7. FABP4 and Plin 1 expressions on day 3 and day 7 were especially upregulated compared to those on day 1, suggesting a rapid lipid accumulation. Two major adipokines, adiponectin and leptin expressions on day 7 were also significantly upregulated, indicating increases in adipocytes maturity and functionalization (Figure 6(c)). Overall, these results suggested that the cells outgrown from fat organoids still maintained a high adipogenesis potential.

## Materials and methods

### Materials

Thrombin, fibrinogen, and urokinase were purchased from Sigma. Nanoscaffolds were obtained from Reinnervate. All flow cytometry antibodies were from BD Biosciences. RNA extraction reagent, the glycerol colorimetric assay kit, and Hoechst 33,258 were from Applygen Technologies.

### Animals

Sprague-Dawley male rats received a regular diet and were sacrificed at age 5–6 weeks on demand. Animal experiments were performed according to guidelines of the US National Institutes of Health guidelines and were approved by the Institute Animal Care and Use Committee of Peking University Health Science Center.

### Whole-mounted superficial fascia and histochemical staining

According to our laboratory-established method [13,18,19], whole-mounted superficial fascia was adhered to the glass slides and fixed by 4% paraformaldehyde for 0.6 h at room temperature. For conventional staining, fascial adipocytes were stained with Oil-red O (0.5%) for 20 min or Nile Red (1 μg/mL) for 10 min. For immunohistochemical staining, fascia tissues were treated with Triton X-100 (0.2%), blocked in defatted bovine serum albumin (1%) for 60 min, stained with antibodies to Plin1, CD29, and CD24 at 4°C overnight, then probed with IgG conjugated to FITC for another 40 min at 37°C. After staining with Hoechst 33,258 for 5 min, fascia sheet was washed twice. Images were captured by the confocal microscopy (Leica TCS SP8).

### Isolation and adipogenic differentiation of fascia-derived stromal cells (FSCs)

FSCs were isolated in accordance with our previously established methods [13,18,19]. Fascia was minced into 1- to 3-mm^3^ pieces, digested by type I collagenase (0.8 mg/mL) at 37°C, then shaken for 2 h at 140 cycles/min. The enzymatic digestions were filtrated with 0.15 mm mesh and centrifuged at 2500 rpm for 15 min. FSC pellets were resuspended in high glucose (4.5 g/L) DMEM containing 10% foetal bovine serum (FBS). FSCs emerged confluent after culture for 4 days and then were treated with adipogenic inducers (250 mM isobutylmethylxanthine, 10 mg/mL insulin, and 0.1 mM dexamethasone) in DMEM with 10% FBS for 48 h. Then, the cells were cultured for another 4 days in high-glucose (4.5 g/L) DMEM containing 10% FBS and 5 mg/mL insulin.

### 3D tissue culture of superficial fascia fragments on fibrin hydrogel

The superficial fascia tissues situated between the hindlimb skeletal muscles and subcutaneous dermis of rats were lifted up with tweezers and cut off using surgical scissor, and washed with DMEM under sterile conditions. Then, the superficial fascia sheets were minced into 1- to 3-mm^3^ sized fragments (Figure 2(b,c)), suspended in a fibrinogen (Sigma) solution dissolved in DMEM at a concentration of 5 mg/mL. This fascia fragment suspension was mixed with the same volume of DMEM containing thrombin (1 U/mL, Sigma) and CaCl_2_ (40 mM), and this mixture was transferred into a culture plate with a radius of 7.73 mm. The concentrations of thrombin and fibrinogen were 0.5 U/mL and 2.5 mg/mL in the final hydrogel system, respectively. The average time for initial gelation of the 3D fibrin hydrogel was 30 to 60 min, and 3 to 5 h was needed for complete polymerization in a humidified chamber at 37°C, which was translucent and had a thickness of 5.24 mm (Figure 2(e–g)). After complete polymerization, the growth culture medium composed of high-glucose (4.5 g/L) DMEM with 10% FBS was added to culture plate to cover 3D hydrogel-encapsulated superficial fascia fragments (Figure 2(h,i)) to sustain cell growth. The culture medium was changed every 2 days. For adipogenic induction, hydrogel was induced on day 6 for 48 h with inducers (250 mM isobutylmethylxanthine, 10 mg/mL insulin, 0.1 mM dexamethasone). Except for special instructions, adipogenic inducers were used in the following experiments. After 30 days, the outgrown cells in the 3D fibrin hydrogels were recovered with 1,000 U urokinase (Sigma) for 60 min along with 10% FCS at 37°C. Next, recovered outgrown cells were cultured in a conventional 2D culture.

### Flow cytometry analysis

Cells outgrown from fat organoids were isolated, cultured, and harvested at 80% confluence. Cells were incubated in Pharm Lyse at 4°C for 15 min and resuspended in Pharmingen stain buffer (BD Biosciences). The outgrown cells were immunostained with antibodies against adipose progenitor markers [25], such as CD29, CD44, CD90, CD24, and CD106; or endothelial marker CD31; or haematopoietic marker CD45, at 4°C for 40 min. After incubation, the outgrown cells were washed with phosphate buffered saline three times and analysed by flow cytometry (Beckman).

### Histology and phenotype of the outgrown cells in the hydrogel

*In vitro* 3D-cultured fascia fragments including outgrown cells and hydrogel were fixed in 1% paraformaldehyde for 4 hours at 4°C, paraffin-embedded and sectioned. For routine histological staining, paraffin section was stained with haematoxylin-eosin (H&E). Elastic-collagen fibre was stained using Victoria Blue (0.5%) and Ponceau S (0.5%). For immunostaining, sections were stained using antibodies specific for proliferating nuclear cell antigen (PCNA); the stromal adipogenic progenitor markers CD29, CD90, CD106, CD44, and CD24; pericyte markers α-SMA, CD146 and PDGFRβ; endothelial markers CD31 and CD34; haematopoietic marker CD45. Antibodies are shown in Online Supplemental Table S1.

### Detection of proliferative outgrown cells

To detect proliferative outgrown cells, an *in vitro* label-retaining assay was used, adding 20 μM 5-ethynyl-2-deoxyuridine (EdU) to the culture system on day 6 for 10 h. Then, the 3D constructs were sampled and captured by two-photon fluorescence microscopy. The EdU assay was conducted in accordance with the manufacturer instructions (RiboBio).

### Adiponectin and leptin secretion in medium

Adipokines secretion in the conditioned medium secreted by the superficial fascia fragments in hydrogel with adipogenic induction were collected at day 9, 15 and 30 and stored at −80°C until further analysis. Adiponectin (Boster) and leptin (ABclonal) levels were assayed by enzyme-linked immunosorbent assay (ELISA) kits in accordance with manufacturer’s protocol. Every independent experiment was repeated at least three times, and quantitative data are expressed as mean ± SEM.

### Lipolysis assay

Adipocyte triglyceride is hydrolysed to glycerol and fatty acids. Glycerol content was measured as an indicator of lipolysis reaction [19,28]. 3D constructs were washed and incubated with Krebs-Ringer solution including 200 nM adenosine, 1% defatted albumin, and 25 mM HEPES, with or without isoproterenol. Glycerol release was quantified by the enzyme-coupled GPO-Trinder reactions at 550 nm absorbance [19] by using a glycerol colorimetric assay kit (Applygen Technologies).

### RNA isolation and quantitative real-time PCR

Total RNA was extracted with RNA extraction reagents (Applygen Technologies). Reverse transcription involved using random primers with the Revert Aid First Strand cDNA Synthesis Kit (Thermo) and quantative RT-PCR (qRT-PCR) involved using the GoTaq qPCR Master Mix (Promega). Oligonucleotide primers were designed with Ventor’s software. All reactions were performed for three times and the values were normalized to 18S RNA level. Primers are shown in Online Supplemental Table S2.

## Statistics

Differences between groups were analysed by using conventional Student’s *t* test or ANOVA. Each experiment was repeated at least three times, and quantitative data are expressed as the mean ± SEM.

## Discussion

This report describes an adipose-tissue organoid originating from non-adipose superficial fascia. Such fascia-originated fat organoid has a typical structure of adipose tissues and possess the principal function of adipose cells in the synthesis, storage, and hydrolysis of triglycerides and adipokines secretion. Most significantly, the generation of fascia-derived fat organoids strongly supports that adipose cell and adipose tissue can originate from the superficial fascia, a non-adipose tissue. This finding could reveal that non-adipose fascia participates in the neogenesis or formation of adipose cells and hence the regulation of adipose metabolism, novel functions of the fascia that have not yet been revealed by classical histoanatomy.

Organoids are large 3D cellular aggregates formed by various tissue or cell sources, such as primary tissue and cells, cell lines, and adult and pluripotent stem cells [2]. According to the discussion of previous reports [3,29,30] and the common viewpoints of histology and physiology, we would suggest that a successful organoid should fulfill the following five basic criteria: 1) a definite volume and generally maintaining a long-time survival for 2 to 4 weeks *in vitro*; 2) regular and organized histological and cytological structures similar to the original organ; 3) the presence of stem cells inside organoids; 4) isolated cells outgrown from the organoid to be capable of the potential differentiation into organ-specific cell types; and 5) the essential functions of their original organ, such as fundamental physiological and biochemical functions. According to these criteria, currently, such as retina, brain, prostate, stomach, kidney, lung, pancreas, liver, small intestine, and large intestine organoids, have been successfully developed *in vitro* and are generally accepted [2–4].

Recently, the fabrication of adipose-tissue organoids has been a crucial concern. Studies in 3D culture of preadipocytes are not rare, and some claim that adipoid-tissue has been built. However, to the best of our knowledge after careful review of the published reports, none of fat or fat-like organoids reported could fully meet the five criteria mentioned above. Mauney et al. used various matrices and scaffolds to support 3D culture of bone-marrow-derived stem cells and adult adipose-derived stromal cells (ASCs), which differentiated into adipocyte-like cells with numerous lipid droplets smaller than typical unilocular adipocytes *in vivo*. When transplanted into skeletal muscle of nude rats for 4 weeks, ‘adipose-like tissue’ did not exhibit the characteristics of adipose tissue or typical mature adipocytes [31]. Similarly, Paek et al. constructed a microengineered 3D culture system for co-culture of human ASCs and adipose microvascular endothelial cells, by inducing adipogenesis and vasculogenesis simultaneously for 40 days. The authors observed a large number of small lipid droplets with sporadic distribution, different obviously from the appearances of classic unilocular adipocytes, although they declared successful construction of vascularized human adipose tissue *in vitro* [9]. Chun et al. showed that the type I collagen component of 3D matrigel may affect the adipogenic differentiation of ASCs and found that as compared with 2D culture, adipocytes had slightly more and larger lipid droplets in matrigel but still no unilocular adipocyte-like appearance [10].

Superficial fascia and all other types of fasciae are continuous framework of connective tissue originating from mesoderm. Although the histology of all types of fasciae is relatively simple and similar, as we discussed previously [13], their intrinsic features and appearances, such as fascial fibres and matrix substance, cells, blood and lymph and nervous vessels actually vary largely depending on species, anatomic regions, and the concrete organs or tissues where fasciae are localized. Consequently, it is not surprising that the fascial progenitors, or so-called mesenchymal cells, should be also varied in stem cell types and markers, cell yield or differentiation potential, depending on species, and specimen sources of different anatomic regions of the body. For example, the mouse superficial fascial cells only had low adipogenic potential or require special inductive cues, significantly different from those of humans, porcine and rats [13,16]. Even, in contrast with the stromal cells of superficial fascia in rats, cells derived from rat visceral fasciae of different internal organs were moderately or very poorly differentiated into adipocytes, likely owing to fewer adipogenic progenitors in such fascia capsules featured by barren vasculatures but dense fibres [13]. In comparison, fibro-adipogenic progenitors (FAPs) resident in the interstitium fascia of skeletal muscle were multipotent progenitors with surface marker PDGFRα and could differentiate into adipocytes, fibroblasts, osteoblasts and chondrocytes [32,33]. Other investigators reported that PDGFRα+ progenitors residing outside of blood vessel, an adventitia fascia we thought, could be a progenitor source for postnatal WAT development but not adult WAT homoeostasis [34]. These types of fascia-related adipogenic progenitors [32–34] have varied features, different with the linage-committed preadipocytes of rat superficial fascia, as we identified in the previous [13] and present studies, which shared multiple surface markers of stromal adipogenic precursors, capable of differentiating into adipocytes, but not into myocytes or osteocytes [13]. Likely, adipogenic progenitors from superficial fascia and other types of fasciae exhibit functional and differentiation heterogeneities, which might be attributed at least in part, fascia type and mesenchymal stem cell type and their niche microenvironment, vasculatures or nutrient supply.

Considering high adipogenic differentiation potential of rat superficial fascia [13], which can avoid the brittleness and floatability of adipose tissue and is easily separated, it is an ideal material for investigating non-dipose-derived preadipocytes capable of adipogenic differentiation in the 3D hydrogel. Cells outgrown from superficial fascia proliferate rapidly in the hydrogel, and were able to differentiate into organized adipocyte aggregates, a fat organoid. Nearly 50% of outgrown cells expressed PCNA, and 50% to 80% incorporated EdU in organoids, suggesting that most of the outgrown cells were derived from *in vitro* cell proliferation and division, not from simple *in situ* migration of resident cells in fascia fragments. Superficial fascia in 3D fibrin were able to differentiate into unilocular adipocytes, and transformed into organized adipocyte aggregates, a fat organoid, which had adipogenic progenitors inside.

Significantly, outgrown cells from superficial fascia fragments in the 3D hydrogel expressed a considerable amount of stromal progenitor cells markers, such as CD29, CD90, CD106, and CD44, known markers of adipogenic precursors within adult adipose tissues [25]. The expression of the pericyte markers α-SMA, CD146 and PDGFRβ was relatively low, with almost no expression of the haematopoietic and vascular endothelial markers CD45, CD31, and CD34, perhaps because of relatively poor vascularity, which was consistent with our previous findings [13]. Choi et al. reported a 3D culture of the selectively dissected connective tissues surrounding peri- or inter-skeletal muscle and adipose tissue, and showed that cells outgrown from the muscle epimysial, deep and superficial fascia expressed the stromal progenitor and pericyte markers CD90, CD44, CD29, α-SMA, and PDGFRβ, and were able to differentiate into adipocytes [16], in accordance with our observations. In particular, the outgrown cells showed a typical differentiation potential of adipogenic lineages. Overall, we successfully constructed fascial fat organoids, which contained typical unilocular adipocytes, adipogenic stem cells, and spatially organized 3D adipocyte architectures; these organoids exhibited the principal function of adipose cells in the synthesis, storage, and hydrolysis of triglycerides.

In comparison, neither subcutaneous nor visceral adipose tissue fragments can successfully differentiate into and form fat organoids in our hydrogel system. The cells outgrown from *in situ* adipose tissues were physiologically inactive, which may be interrupted by the oil floating on the upper layer of culture medium and affecting nutrition and gas exchange (data not shown). This fibrin hydrogel seemed also not suitable for primary stromal cells isolated because collagenase for digesting tissues always destroyed the 3D structure, although Suelzu et al., using passage ASCs as original materials, reported that fibrin could support 3D cell culture [35,36]. Primary FSCs were not successful in fibrin culture systems because using collagenase might result in the collapse of collagen networks. We used nanoscaffolds to support the 3D culture of FSCs to overcome this limitation, and they showed numerous adipocyte-spheroids featuring a variable number of cytoplasmic lipid droplets but not unilocular adipocytes, not even fat organoids (Figure S2). Fewer vascular networks and poor nutrition supply of 3D nanosystems may be one of the main reasons for this finding.

Notably, fascia originated fat organoids represent a more physiological system for studying adipogenesis *ex vivo* or *in vitro*, and might have broad prospects for applications in large-scale drug screening, plastic surgery, and regenerative medicine. With histological point of view, fascia, a non-adipose tissue, can generate adipose tissues. As speculated, humanized 3D constructs could become accessible on the basis of systematic characterizations of the fascial fat organoid derived from rats. Here, we provide evidence for the fascial origin of fat organoids, which may strengthen our understanding of metabolic regulation by crosstalk between multiple different organs and tissues and provide new therapeutic strategies for metabolic diseases.

The construction of organoids should be carried out in a manner that could mimic the *in vivo* physiological environment as possible. Further refinements of the 3D culture system are necessary. A variety of growth factors and microvessel fragments are crucial for adipogenesis [37,38], which may benefit 3D tissue engineering. We found that epidermal growth factor (EGF) could significantly stimulate cell growth and proliferation, but those were not beneficial to the formation of fat organoids (data not shown), consistent with a previous report that epidermal growth factor inhibited adipocyte differentiation in a 2D system [39]. Suresh et al. showed that vascular endothelial growth factor could induce an endothelial-cell phenotype of adult ASCs and promote the formation of tubules within 3D matrigel [40]. Strobel et al. used microvessels isolated from adipose tissue as the source of the vascular networks, which could improve the function of vascularized adipocyte organoids by increasing insulin receptor expression and altering responsiveness to an inflammation challenge [38]. Therefore, we could attempt to add vascular endothelial growth factor or microvascular fragments into the 3D system and further optimize the formation of fat organoids derived from fascia because of the poor vascularity of the current system, and to acquire a more stable mature adipocyte phenotype with better functionality, longer survival time, and larger size of fat organoids.

## Conclusions

We established a fascia-originated 3D fat organoid with the typical structure of adipose tissue and cells and possessed the principal function of adipose cells in the synthesis, storage, and hydrolysis of triglycerides. Fat organoids originating from fascia provide a new approach for adipocyte research, and may have broad prospects for their application in regenerative medicine and plastic surgery, and may also be used for building fat organoid-based bioreactors when combined with microfluidic techniques. Most importantly, fascia fat organoids could provide strong evidence showing that both adipose tissues and cells can originate from fascia, a non-adipose tissue. Our findings may provide insights into metabolic regulation by the crosstalk between multiple different organs and tissues and new knowledge for investigating novel treatment strategies for obesity or other related metabolic diseases.

## Supplementary Material

Supplemental Material

## Data Availability

The data that support the findings of this study are openly available in ‘figshare’ at https://doi.org/10.6084/m9.figshare.19395119. More details of this study is available from the corresponding author upon reasonable request.
