# Supplementary material for: Generation of functional fat organoid from rat superficial fascia
Source: Adipocyte. 2022 May 13;11(1):287–300. doi: 10.1080/21623945.2022.2072446 (PMC9116422; doi:10.1080/21623945.2022.2072446)
Supplement: Supplemental Material [file KADI_A_2072446_SM1281.docx]

**Supplementary data**

**Table S1. Antibodies used for immunohistochemical staining**

| **Antibody** | **Vendor** |
| --- | --- |
| Perilipin-1 | Gifts from laboratory of C. Londos at the U.S. National Institutes of Health |
| Perilipin-2 | Gifts from laboratory of C. Londos at the U.S. National Institutes of Health |
| PCNA | Abcam |
| CD44 | Invitrogen |
| CD24 | Abcam |
| CD29 | Invitrogen |
| CD90 | Abcam |
| α-SMA | Cell Signaling |
| PDGFRβ | Abcam |
| CD106 | Abcam |
| CD31 | Sigma |
| CD45 | Sigma |
| CD24 | Invitrogen |
| CD34 | Invitrogen |

**Table S2. Primers used for quantitative real-time PCR**

| **Gene** | **Forward sequence (5’-3’)** | **Reverse sequence (3’-5’)** |
| --- | --- | --- |
| PPARγ | CCGAGAAGGAGAAGCTGTTG | TCAGCGGGAAGGACTTTATG |
| FABP4 | AGCCCAACTTGATCATCAGC | TCCTGTCATCTGGGGTGATT |
| Plin1 | TGCGCAAGAAGAGCTGAGTA | AGAGGCCAACCTGAAGGAGT |
| FAS | AAGCCCTTGGGAGTCAAAGT | TAGACGTCAGCAGGTCGATG |
| CEBPα | ATCCCAGAGGGACTGGAGTT | TTTAGCATAGACGCGCACAC |
| AdipoQ | TATCACTCAGCATTCAGCGTAG | AAGAGGCTCACTTTCACATCC |
| Leptin | GTTCCTGTGGCTTTGGTCCT | GGACAAACTCAGAATGGGGTG |
| 18S rRNA | CGCTAGAGGTGAAATTCTTG | GGAACTACGACGGTATCTGA |

**Table S3. Cytological phenotypes of the cells outgrown from fat organoids**

| **Markers** | **Percentage of adipose progenitors (%)** |
| --- | --- |
| CD29 | 99.21 ± 0.21 |
| CD44 | 63.06 ± 4.72 |
| CD90 | 90.96 ± 0.13 |
| CD24 | 5.21 ± 0.16 |
| CD106 | 53.74 ± 0.22 |
| α-SMA | 30.26 ± 3.45 |
| PDGFRβ | 43.64 ± 1.12 |
| CD45 | 0.24 ± 0.19 |
| CD31 | 0.13 ± 0.22 |


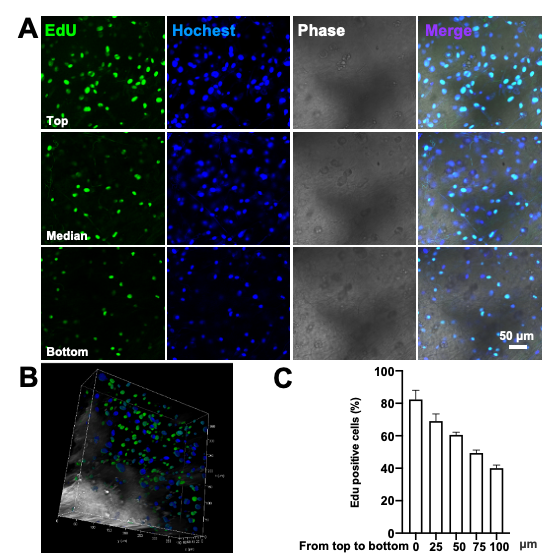


**Figure S1. Proliferation of outgrown cells in the 3D fibrin hydrogel.** (A) Two-photon fluorescence imaging of EdU-labeled proliferative cells at the top (0 μm), median (50 μm), and bottom (100 μm) scanning depths of hydrogel scaffolds. More than 50% to 80% of the outgrown cells expressed EdU. (B) 3D reconstructed image with a width of 400.05 μm, height of 400.05 μm, and depth 110.50 μm. Proliferative cells and nuclei were stained by EdU (green) and Hoechst 33258 (blue), respectively. (C) Quantification of EdU-positive outgrown cells from the upper layer of the fibrin matrix and below. Data are mean±SEM. EdU, 5-ethynyl-2-deoxyuridine.


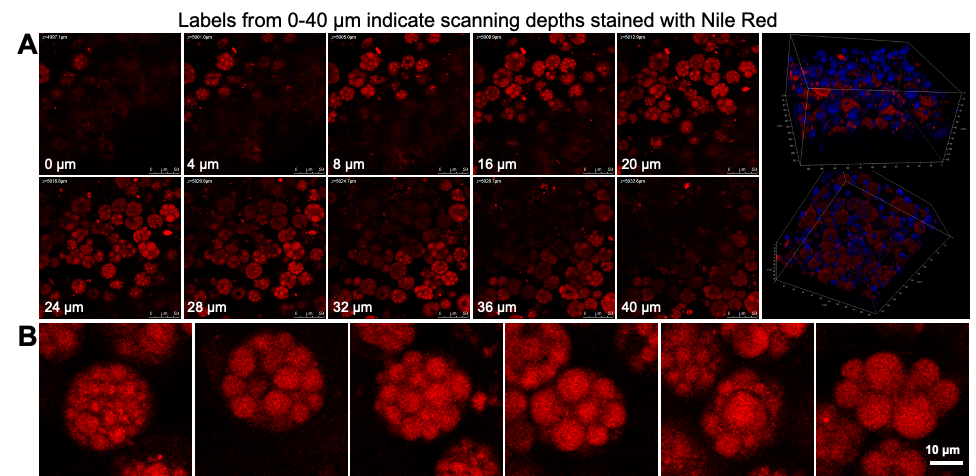


**Figure S2: Nano scaffold-supported 3D culture of fascia-derived stromal cells (FSCs) for 30 days.** (A) Nile Red staining of FSCs in nanoscaffold. Two-photon fluorescence views and 3D reconstructed imaging (width 220.05 μm, height 220.05 μm, depth 120.50 μm) showed numerous differentiated adipocytes containing cytoplasmic lipid droplets in scaffolds with a scanning depth of 0 to 40 μm. (B) Multiple adipocyte-spheroids but not fat organoids were formed.
